# Models based on nucleic acid methylation regulators will contribute to facilitating cancer precision medicine

**DOI:** 10.1186/s12916-021-02171-6

**Published:** 2021-12-01

**Authors:** Wen Zhang

**Affiliations:** Department of Immunology, National Cancer Center/National Clinical Research Center for Cancer/Cancer Hospital, Chinese Academy of Medical Sciences and Peking Union Medical College, Beijing, 100021 China

**Keywords:** 5mC, m^6^A, Methylation regulator, Bladder cancer, Small-cell lung cancer, Immunotherapy response, Precision medicine

## Background

With the development of molecular biology techniques, it is possible to explore the tumorigenesis and cancer development mechanisms and intrinsic characteristics of single cancer types from the perspectives of chromosome, genome, transcriptome, protein expression, and epigenetics, and on this basis, molecular subtypes can be classified [1, 2]. Epigenetics is generally considered as chemical modification, including DNA and RNA methylation, non-coding RNA modification, histone modification, and chromatin rearrangement [3]. In recent years, nucleic acid methylation modification and regulators have become a hot topic in cancer research, along with showing potential clinical application in predicting immunotherapy response and prognosis [4].

## 5-Methylcytosine (5mC) regulator in predicting immunotherapy response in bladder cancer (BLCA)

5mC plays an important regulatory role in tumorigenesis and development, and the discovery of 5mC regulators further indicate that DNA methylation plays an important role in maintaining genomic stability, mediating tumor cell differentiation, and shaping the tumor microenvironment (TME) [5]. Hu et al. [6] report a novel 5mC regulator-mediated molecular subtype system to predict the classical molecular subtypes, immunophenotypes, and clinical outcomes of BLCA. This study established a 5mC score system according to mRNA expression level, and BLCA patients were characterized into two subgroups according to the score. To note, because the mRNA expression and a high copy number amplification rates of genes (including *MDM2*, *MDM4*, *DNMT3A*, *CCND1*, *FGF3*, *FGF4*, and *FGF19*) were positively associated with immune-checkpoint blockade (ICB) response and were significantly higher expressed in the high 5mC score group, the incidence of ICB-related hyperprogression may be higher in the high 5mC group. This finding appears to be relevant for clinical treatment response. First, the 5mC score was negatively correlated with the anti-cancer immunity, and a high 5mC score is negatively related to the response to ICB and a higher incidence of ICB-associated hyperprogression. Second, the 5mC score was significantly related to the cancer stemness and could reflect TME heterogeneity. Usually, a higher cancer stemness indicates a decrease in anti-cancer immunity and lower ICB response. Thus, the 5mC score could effectively stratify the immune phenotypes of BLCA. In addition, the 5mC score was able to predict the clinical response to other treatments, including EGFR targeted therapy, radiotherapy, and several therapies targeting immune inhibited oncogenic pathways.

BLCA is one of the most common urinary malignancies. Despite great advances in ICB, neoadjuvant chemotherapy, and targeted therapies, many patients with advanced BLCA are not sensitive to these therapies, and there are no reliable and effective biomarkers or tools to accurately predict the clinical response to these therapies. This study demonstrates that the 5mC regulators-based subtype system could reflect many aspects of bladder cancer biology and provide new insights into bladder cancer therapy.

## N6-methyladenosine (m^6^A) regulator in predicting immunotherapy response in small-cell lung cancer (SCLC)

m^6^A is one of the most common post-transcriptional modification of eukaryotic mRNA [7]. The effect of m^6^A in cancer is reflected in the regulation of cancer-related gene expression. m^6^A affects the occurrence and development of cancer by enhancing or inhibiting the expression of oncogenes and tumor suppressor genes and plays different roles in different tumors and different pathways [8]. In a recent study by Zhang et al. [9], the importance of m^6^A modified regulators in small cell lung cancer was illustrated, and a prognostic feature based on multicentric m^6^A regulators was developed for limited-stage SCLC (LS-SCLC) patients. This study identified that there may be a large number of m^6^A modifications in LS-SCLC, and its dysregulated expression may be involved in the occurrence and development of SCLC. Using LASSO Cox model, 5 significant candidates (*G3BP1*, *METTL5*, *ALKBH5*, *IGF2BP3* and *RBM15B*) among 22 m^6^A regulators were selected and used to establish a m^6^A score system. The authors report m^6^A score was an independent prognostic predictor in LS-SCLC. One of the interesting findings is that the m^6^A score is significantly associated with therapeutic response and clinical benefit in both patients receiving adjuvant chemotherapy (ACT) and anti-PD-1 immunotherapy. Patients with low scores received a greater survival benefit from ACT, exhibited more CD8+ T cell infiltration, and were more responsive to ICB.

In recent years, with the focus on the role of m^6^A in cancer, increasing evidence suggest that m^6^A regulators are promising prognostic biomarkers which help determine chemotherapy and immunotherapy resistance [10]. The excellent work by Zhang et al. showed a new application of m^6^A regulators in cancer treatment response. Although there may inevitably be some deviations in the analysis of this study, more prospective and reliable studies are needed. This study is the first systematic examination of the m^6^A modification pattern in LS-SCLC to establish a comprehensive prognostic model of m^6^A regulators. The large study cohort increases the reliability and robustness of the m^6^A score model. Furthermore, the m^6^A score model based on tissue samples to predict the benefit of chemotherapy and immunotherapy has potential clinical application in the treatment and clinical management of SCLC patients.

## Conclusions

The studies by Hu et al. and Zhang et al. suggest that the regulation of nucleic acid methylation is an important part of tumor research. It not only explains the characteristics of tumor epigenetics but also provides a potential molecular biological mechanism for tumorigenesis and development, anti-tumor immune response, and therapeutic response. The establishment of the prediction model for nucleic acid methylation regulators can effectively supplement the evaluation of clinical responses. Although limitations like the need for further validation in multiple centers exist, these studies can provide a strong foundation for precision medicine.

## Data Availability

Not applicable.

## Abbreviations

5mC: 5-Methylcytosine
BLCA: Bladder cancer
TME: Tumor microenvironment
ICB: Immune-checkpoint blockade
m^6^A: N^6^-methyladenosine
SCLC: Small-cell lung cancer
LS-SCLC: Limited-stage SCLC
ACT: Adjuvant chemotherapy
